# Prognostic models for predicting incident or recurrent atrial fibrillation: protocol for a systematic review

**DOI:** 10.1186/s13643-019-1128-z

**Published:** 2019-08-28

**Authors:** Janine Dretzke, Naomi Chuchu, Winnie Chua, Larissa Fabritz, Susan Bayliss, Dipak Kotecha, Jonathan J. Deeks, Paulus Kirchhof, Yemisi Takwoingi

**Affiliations:** 1 Institute of Applied Health Research, University of Birmingham, Edgbaston, Birmingham, B15 2TT UK; 2 Institute of Cardiovascular Sciences, University of Birmingham, Edgbaston, Birmingham, B15 2TT UK; 3 UHB NHS Foundation Trust, Birmingham, UK; 4 SWBH NHS Trust, Birmingham, UK

**Keywords:** Atrial fibrillation, Incidence, Recurrence, Prognostic model, Risk score, Model validation, Model performance, Systematic review

## Abstract

**Background:**

Atrial fibrillation (AF) is the arrhythmia most commonly diagnosed in clinical practice. It is associated with significant morbidity and mortality. Prevalence of AF and complications of AF, estimated by hospitalisations, have increased dramatically in the last decade. Being able to predict AF would allow tailoring of management strategies and a focus on primary or secondary prevention. Models predicting recurrent AF would have particular clinical use for the selection of rhythm control therapy. There are existing prognostic models which combine several predictors or risk factors to generate an individualised estimate of risk of AF. The aim of this systematic review is to summarise and compare model performance measures and predictive accuracy across different models and populations at risk of developing incident or recurrent AF.

**Methods:**

Methods tailored to systematic reviews of prognostic models will be used for study identification, risk of bias assessment and synthesis. Studies will be eligible for inclusion where they report an internally or externally validated model. The quality of studies reporting a prognostic model will be assessed using the Prediction Study Risk Of Bias Assessment Tool (PROBAST). Studies will be narratively described and included variables and predictive accuracy compared across different models and populations. Meta-analysis of model performance measures for models validated in similar populations will be considered where possible.

**Discussion:**

To the best of our knowledge, this will be the first systematic review to collate evidence from all studies reporting on validated prognostic models, or on the impact of such models, in any population at risk of incident or recurrent AF. The review may identify models which are suitable for impact assessment in clinical practice. Should gaps in the evidence be identified, research recommendations relating to model development, validation or impact assessment will be made. Findings will be considered in the context of any models already used in clinical practice, and the extent to which these have been validated.

**Systematic review registration:**

PROSPERO (CRD42018111649).

## Background

Atrial fibrillation (AF) is the most common arrhythmia diagnosed in clinical practice, and the worldwide incidence and prevalence are increasing [1]. AF is predicted to affect around 18 million people in Europe by 2060, and 6–12 million in the USA by 2050 [1, 2]. Drivers for the increase in prevalence include an ageing population, better survival from conditions such as ischaemic heart disease, but also increasing multimorbidity [3, 4]. AF is associated with increased morbidity, such as stroke or heart failure, and increased mortality, particularly cardiovascular related [3, 4]. Currently available treatments can reduce the mortality and morbidity associated with AF, particularly via anticoagulation for prevention of strokes [4], but patients with AF remain at high risk of cardiovascular complications even on optimal therapy, often manifesting as heart failure or sudden death [5]. AF also commonly occurs after cardiac surgery, with patients subsequently at increased risk of stroke, congestive heart failure, and haemodynamic instability [6]. Post-operative AF rates have been estimated at up to 60% for cardiothoracic surgery and 5–10% in non-cardiothoracic surgery [7].

Given the growing burden of AF, there has been a recent shift in attention to primary and secondary prevention strategies [8, 9]. It has long been known that approximately 70% of patients experience recurrent AF after, e.g. a cardioversion [10], while a small population of AF patients does not show progression of AF, with rare recurrences over decades [11]. Being able to identify those patients who are most likely to develop recurrent AF, or progress from paroxysmal to sustained AF, would be beneficial for tailoring treatment strategies and implementing targeted preventative measures. There is a range of risk factors associated with the development of AF, the best-established ones being age, hypertension, diabetes mellitus, and heart failure, as well as environmental factors such as smoking and alcohol consumption [12, 13]. Less validated risk factors include subclinical hyperthyroidism, obesity and sleep apnoea syndrome [12]. There may also be a role for biomarkers in assessing AF risk, including serum biomarkers such as brain natriuretic peptide (BNP) [14, 15] or fibroblast growth factor 23 (FGF-23) [14], imaging of atrial function, ECG-based parameters, and genetic factors [12].

A number of prognostic models, or risk scores, which attempt to combine several predictors to generate an individualised estimate of risk in different populations have been developed for AF prediction. These include the Cohorts for Ageing and Research in Genomic Epidemiology (CHARGE)-AF score [16], the HAVOC score [17], the ATLAS score [18], the HATCH score [19], and the APPLE score [20].

Scoping searches for existing and ongoing systematic reviews of prognostic models for AF were carried out in MEDLINE, Embase, the Cochrane Library, and PROSPERO (October 2018). There were several systematic reviews relating to either the association of individual risk factors with AF or focussing on a single AF risk prediction model; only one identified systematic review gave an overview of available risk scores; this had a focus on recurrence following catheter ablation [21]. The review had a limited search strategy and did not include formal quality appraisal of the identified models. More recent primary studies were identified since the publication of this review. There is therefore a need for an up-to-date systematic review, which includes all relevant populations and uses methodologically robust methods for study identification, risk of bias assessment, and synthesis.

### Aim

To undertake a systematic review of prognostic models predicting incident or recurrent AF, with meta-analysis of model performance measures where possible.

More specifically to

- Identify all studies which report on the development, validation, or impact of a score for predicting AF
- Take forward for risk of bias assessment and analysis, all studies reporting validated models (internal and/or external validation)
- Summarise and compare model performance measures and predictive accuracy
- Undertake meta-analysis of model performance measures where possible

## Methods

### Study eligibility criteria

#### Study design

Studies of any design reporting the following types of prognostic modelling will be eligible for inclusion as guided by the CHARMS [22] checklist:

- Prediction model development with internal validation
- Prediction model development with external validation
- External model validation (with or without model updating)

Studies reporting impact assessment of a prognostic model will also be eligible for inclusion.

Studies that have developed a model but not validated this will not be taken forward for risk of bias assessment and analysis, but a record of these studies will be kept.

For the purpose of this review, a prognostic model will be defined as a combination of two or more predictors within a statistical model, which is used to predict an individual’s risk of the outcome [23]. Published and unpublished studies, as well as studies published in abstract form only, will be eligible for inclusion. Studies that have looked at the association between a single risk factor and the development or recurrence of AF will be excluded, as they are limited in their utility for individual risk prediction [24].

#### Patient group

Any population at risk of incident or recurrent AF will be eligible for inclusion. This will include the general population; people with cardiovascular disease or other comorbidities; people who have undergone surgery and who may be at risk of post-operative AF; and people who have been treated for AF and are at risk of recurrent AF. There will be no restriction on the number or type of previous treatments. People with paroxysmal, persistent, or permanent AF will be eligible, and in this case, models predicting progression from one type of AF to another will be of interest.

#### Outcomes

The clinical outcome of interest is incident or recurrent AF and/or progression from paroxysmal to persistent or permanent AF. Model performance will be assessed by calibration measures, i.e. how well the predicted risks compare to the observed outcome, and discrimination measures, i.e. how well the model differentiates between those with and without the outcome [25].

### Search strategy

The following bibliographic databases will be searched (from inception to November 2018) using combinations of text and index terms relating to the incident or recurrent AF and models: MEDLINE (Ovid), MEDLINE In-Process (Ovid), Embase (Ovid), and Cochrane CENTRAL. The terms relating to the ‘model’ component of the search strategy will be informed by the updates to two existing study design filters as described by Geersing (2012) [26]. Several alternate terms will be used, as a prognostic model may also be described as a prognostic (or prediction) index or rule, risk (or clinical) prediction model, or predictive model [23]. The term ‘clinical score’ will also be used as well as names of known scores identified through scoping searches. Searches will be updated shortly before project completion to ensure the systematic review is up-to-date at the time of submission. See Appendix for sample search strategy in Embase.

There will be no date or language restrictions. Reference lists of included articles and relevant reviews will be checked and subject experts consulted. ClinicalTrials.gov and the WHO International Clinical Trials Registry Platform will be searched for ongoing studies. The Conference Proceedings Citation Index will be searched for conference abstracts.

### Study selection

All identified studies will be screened independently by two reviewers (JD, NC) using predefined screening criteria, with disagreements resolved through discussion or referral to a third reviewer (YT). A sample of records will be screened by two reviewers to pilot and amend the screening criteria if necessary, before screening the remainder. Full texts will be screened where necessary. Part or full-text translation will be undertaken where necessary to make selection decisions. The screening process will be facilitated by the use of reference management software (EndNote X7) and the selection process documented using a PRISMA [27] flow diagram.

### Data extraction

Data extraction will be undertaken by one reviewer and checked by a second. A pre-defined and piloted data extraction form (Excel 2016) will be used. Items to data extract will be guided by the CHARMS [22] checklist and will include

- Participants (e.g. eligibility and recruitment method, comorbidities, type and length of treatment for AF, surgery prior to AF)
- Study design (e.g. randomised controlled trial, prospective study, sample size, length of follow-up)
- Candidate predictors (e.g. number and type, method of measurement)
- Outcome measures (e.g. incident or recurrent AF, how diagnosed)
- Model development (e.g. modelling method, method for selection of predictors)
- Model performance (discrimination measures, e.g. c-statistic, and calibration measures, e.g. ratio of observed and expected events (*E/O* ratio)
- Model validation (e.g. method for testing model performance)

### Assessment of risk of bias

The quality of studies reporting the development or validation of a prognostic model will be assessed using the Prediction Study Risk Of Bias Assessment Tool (PROBAST) [28]. This assesses criteria within five domains: participant selection (e.g. were inclusions and exclusions of participants appropriate); predictors (e.g. were predictors measured blind to outcome data); outcomes (e.g. was the same definition used for outcomes in all patients); sample size and patient flow (e.g. was there a pre-specified sample size based on estimated number of outcome events, handling of missing data); and analysis (e.g. was selection of predictors based on univariable analysis avoided).

Should impact studies be identified, then additional quality assessment tools will be used depending on study designs (e.g. Cochrane risk of bias tool [29] for randomised controlled trials).

### Synthesis

All studies reporting validated prognostic models will be narratively described, with key findings tabulated (e.g. predictor variables included in different models, reported predictive accuracy of models). Models relating to different populations will be considered separately. We will compare model performance across different models, taking into consideration the quality of the study and thus the likelihood that model findings are accurate. We will also compare risk factors included in different models in order to identify those that are contributing most to predictive accuracy.

If the same model is validated in several studies, and the same discrimination or calibration statistics are reported (e.g. C-statistic, *E/O* ratio), multivariate random effects meta-analysis to jointly summarise calibration and discrimination to obtain average model performance will be considered. A random effects model is more likely to be suitable as validation studies typically differ in design and case-mix, and meta-analysis should allow for the presence of heterogeneity [25]). The *I*^2^ statistic will be used to estimate the proportion of heterogeneity that is due to between-study variability. Meta-analysis will only be undertaken for groups of studies including similar populations. Exploration of heterogeneity through subgroup analyses or meta-regression is unlikely to be feasible, as a minimum of 10 studies per variable is recommended [25] and scoping searches indicate lower numbers of studies relating to individual models. This also precludes formal exploration of publication bias using funnel plots.

The body of evidence identified will be considered and interpreted in the context of the domains described in the GRADE approach (risk of bias, imprecision, inconsistency, indirectness, and publication bias); however, a formal GRADE score will not be calculated.

### Reporting

PRISMA guidelines [27] will be followed for the reporting of the systematic review.

## Discussion

The increasing burden from AF means that a focus on prevention is becoming more important, and being able to identify patients most at risk of incident or recurrent AF is vital for tailoring management strategies. To the best of our knowledge, this will be the first systematic review to collate evidence from all studies reporting on validated prognostic models, or on the impact of such models, in any population at risk of incident or recurrent AF.

We will use recognised systematic review methods for identifying, appraising, and synthesising the available evidence on existing prognostic models, which will strengthen the robustness of any findings. Publication bias, poor reporting, and extensive heterogeneity are recognised issues in prognostic research [23–25]. Careful consideration of heterogeneity before analysis will ensure that studies are not inappropriately grouped. The potential impact of publication bias on any findings will be discussed, and recommendations made for future reporting of prognostic models if appropriate.

Should one or more well-validated models with high predictive accuracy be identified, then this will be useful for planning future comparative studies on the impact of using such models in clinical practice. The review will also identify gaps in the evidence, i.e. where model validity is poor or where models are lacking for a particular population. Review findings will therefore inform research recommendations relating to model development, validation, or impact assessment. Findings will be considered in the context of any models already used in clinical practice, and the extent to which these have been validated.

## Data Availability

Not applicable

## Appendix

Search strategies Embase and MEDLINE.

1. atrial fibrillation.ti,ab.
2. exp atrial fibrillation/
3. 1 OR 2
4. exp recurrent disease/
5. recur$.ti,ab.
6. exp incidence/
7. inciden$.ti,ab.
8. new onset.ti,ab.
9. new$ diagnos$.ti,ab.
10. progress$.ti,ab.
11. prevalen$.ti,ab.
12. exp prevalence/
13. OR/ 4-12
14. prognos$ model$.ti,ab.
15. exp prognosis/ and exp model/
16. predict$ model$.ti,ab.
17. exp prediction/ and exp model/
18. prognos$ index.ti,ab.
19. predict$ index.ti,ab.
20. risk$ predict$.ti,ab.
21. risk$ model$.ti,ab.
22. predict$ rule$.ti,ab.
23. prognos$ rule$.ti,ab.
24. risk score$.ti,ab.
25. clinical score$.ti,ab.
26. prognos$ risk.ti,ab.
27. decision rule$.ti,ab.
28. model$ score$.ti,ab.
29. model performance.ti,ab.
30. decision model.ti,ab.
31. validation.ti,ab.
32. stratification.ti,ab.
33. ROC curve.ti,ab.
34. exp receiver operating characteristic/
35. discriminat$.ti,ab.
36. c-statistic.ti,ab.
37. "Area under the curve".ti,ab.
38. AUC.ti,ab.
39. Calibration.ti,ab.
40. indices.ti,ab.
41. algorithm.ti,ab.
42. multivaria$.mp.
43. (HATCH adj3 scor$).ti,ab.
44. (ALARMEc adj3 scor$).ti,ab.
45. (BASE-AF2 adj3 scor$).ti,ab.
46. (APPLE adj3 scor$).ti,ab.
47. (CAAP-AF adj3 scor$).ti,ab.
48. (MB-LATER adj3 scor$).ti,ab.
49. (CHARGE-AF adj3 scor$).ti,ab.
50. (HAVOC adj3 scor$).ti,ab.
51. (ATLAS adj3 scor$).ti,ab.
52. (FHS adj3 scor$).ti,ab.
53. (ARIC adj3 scor$).ti,ab.
54. (WHS adj3 scor$).ti,ab.
55. (CHADS2 adj3 scor$).ti,ab.
56. (CHA2DS2-VASC adj3 scor$).ti,ab.
57. (R2CHADS2 adj3 scor$).ti,ab.
58. OR/14-57
59. 3 AND 13 AND 58

## Abbreviation

AF: Atrial fibrillation
